# Dentists Are at a Higher Risk for Oral *Helicobacter pylori* Infection

**DOI:** 10.1155/2020/3945189

**Published:** 2020-07-04

**Authors:** Qian Liu, Yunhan Zhang, Chunmei Xu, Boran Chen, Hao Xu, Yangpei Cao, Tingwei Guo, Yuan Gao, Zhou Zhou, Xuedong Zhou, Xin Xu, Jinzhi He

**Affiliations:** ^1^State Key Laboratory of Oral Diseases & National Clinical Research Center for Oral Diseases, West China Hospital of Stomatology, Sichuan University, Chengdu 610041, China; ^2^Department of Periodontics, West China Hospital of Stomatology, Sichuan University, Chengdu 610041, China; ^3^Department of Pediatric Dentistry, West China Hospital of Stomatology, Sichuan University, Chengdu 610041, China; ^4^Department of Stomatology, The Third Hospital of Peking University, Beijing 100191, China; ^5^Chinese Academy of Medical Sciences Research Unit of Oral Carcinogenesis and Management, West China Hospital of Stomatology, Sichuan University, Chengdu 610041, China; ^6^Department of Cariology and Endodontics, West China Hospital of Stomatology, Sichuan University, Chengdu 610041, China; ^7^Center for Craniofacial Molecular Biology, Herman Ostrow School of Dentistry, University of Southern California, Los Angeles, CA 90033, USA; ^8^Clinical Skills Training Center, West China Hospital, Sichuan University, Chengdu, Sichuan, China

## Abstract

Oral cavity has been taken as one of the major reservoirs for *Helicobacter pylori*, the bacteria responsible for gastric infection and cancers. Dentists are frequently exposed to saliva; thus, theoretically, they are at a higher risk for oral *H. pylori* infection. In the present study, to test this hypothesis and to find out the potential factors associated with the increased risk, a cross-sectional study was carried out on a large scale of dentists (*N* = 90) and nondentist controls (*N* = 110). By using nested polymerase chain reaction to amplify a specific DNA fragment of *H. pylori*, we found 7.27% of saliva samples from the nondentist group and 16.67% of saliva samples from the dentist group were oral *H. pylori* positive, and the difference between groups was statistically significant (*χ*^2^ = 4.292, *p* = 0.038). Importantly, however, after stratifying enrolled subjects with factors which might interfere with the comparison of *H. pylori* detection rate between groups, we still observed a higher *H. pylori* frequency in the dentists than that in the controls in subgroups, including those with good individual hygiene, healthy lifestyle, and physical condition, as well as those living with families to be gastric disease free and not sharing meals with *H. pylori*-positive persons, respectively. Moreover, the frequency of clinical practice per week of the investigated dentists was closely associated with an oral *H. pylori* infection risk. Our data indicates that dentists are at a higher risk for *H. pylori* infection, and intensive attention needs to be paid on this issue.

## 1. Introduction

Since its first isolation by Marshall and Warren from gastric biopsy specimens of patients with chronic gastric inflammation and peptic ulcer, *Helicobacter pylori* (*H. pylori*) has long been regarded as the main etiologic agent for chronic gastritis, type B gastritis, and peptic ulcers [1–3]. Worth still, due to its role in gastric cancers (the cancer considered as the second most frequent cause of cancer death worldwide [4]), *H. pylori* is a type 1 carcinogen identified by the International Agency for Research on Cancer of the World Health Organization [2, 4, 5]. Thus, the prevention of *H. pylori* infection is of great clinical significance.

*H. pylori* was also detected in human organs other than the stomach. After Krajden et al. successfully isolated *H. pylori* from dental plaque and saliva [6], the presence of this bacterium in the human oral cavity has been confirmed by several other studies [7–10]. The oral *H. pylori* is closely associated with oral diseases, such as periodontal disease [11], dental caries [12], recurrent aphtha's stomatitis [13], and mucosal inflammation [14]. In addition, previous studies showed that *H. pylori* colonizing the oral cavity might be also involved in gastric diseases because oral *H. pylori* interfered gastric eradication therapy [15–20]. Concomitantly, gastric *H. pylori*-positive patients were more likely to be oral *H. pylori* positive [21], and identical strains of *H. pylori* in the oral cavity and in the stomach have been isolated [8–10, 22, 23]. Meanwhile, chances of occurrence or recurrence of *H. pylori* stomach infection are more likely to be found among persons who harbour this organism in the oral cavity [24]. All these reports suggest that the oral cavity is a potential reservoir of *H. pylori* before it invades gastric mucosa [7]. Therefore, the oral-oral pathway has been suggested to be among the primary routes of *H. pylori* transmission [25], making infected saliva, dental plaque, debris, and eating devices as major vehicles for *H. pylori* spreading.

Compared to the nondentists, dentists are more frequently exposed to the infected oral contents such as saliva and dental plaque. It is conceivable that dentists are at a higher risk for oral *H. pylori* infection. Therefore, the aim of this study was to compare the detection frequency of oral *H. pylori* between dentists and nondentists and to figure out the potential factors closely associated with the oral *H. pylori* infection risk among dental professionals.

## 2. Materials and Methods

### 2.1. Subjects Enrolling

The study protocol was reviewed and approved by the Institutional Review Board of West China Hospital of Stomatology, Sichuan University (WCHSIRB-D-2015-086). Written informed consent was obtained from all participants before sample collection. In total, 96 dentists and 115 nondentists were randomly enrolled in this study from March 2016 to December 2016. Volunteers in the nondentist control group lived in Chengdu for more than 2 years at the start time point as the dentists did and did not pay a visit to a dental clinic for more than one year. Specifically, participants (1) were free of systematic diseases other than gastric diseases, (2) had no previous periodontal treatment and no antibiotic use within the past 3 months, (3) had normal oral mucosal membranes, (4) had more than 24 teeth and free of dental caries, and (5) for more than 90% checking sites, periodontal tissues showed no clinical signs of inflammation, such as redness, swelling, or bleeding on probing, and were judged to be free of gingivitis or periodontitis. Exclusion criteria included pregnancy, the occurrence of systemic diseases other than gastric diseases, receiving antibiotics, and immunosuppressive drug treatment within the past 3 months. The oral examination was carried by two trained dentists. A kappa test on both caries and periodontal diagnosis was done before sampling to assess the reproducibility of these two examiners, and the kappa values for the diagnosis of both diseases were higher than 0.8, indicating the examination results between the two dentists were reproducible.

### 2.2. Sampling

Volunteers were asked to refrain from food and oral hygiene (i.e., brushing or flossing teeth) for 2 hours before sampling. Volunteers were instructed to expectorate into a sterile cryogenic vial (Corning, NY, USA). About 2 mL of spontaneous, unstimulated whole saliva was obtained from each individual. The saliva was put on ice immediately after sampling, transported to the laboratory within 2 hours, and stored at -80°C before further processing. At the same time, subjects were asked to fill a questionnaire. The information of correlative factors contributing to *H. pylori* infection risk was collected, including demographic and socioeconomic status (i.e., age, sex, education background, residence, number of household members, and working years), lifestyle (i.e., washing hand before eating and after toilet, tooth mug sharing, dishware sharing, picky eating, drinking unboiled water, smoking, and alcohol abuse), medical history (e.g., upper gastrointestinal diseases or family member with gastric diseases), and dental professional-related factors (e.g., using PPE, dental practice frequency per week, and the number of patients treated every half-day).

### 2.3. DNA Extraction

TIANamp Swab DNA Kit (Tiangen Biotech, Beijing) was used to extract genomic DNA following the manufacturer's instruction. The DNA samples were stored at -20°C until use.

### 2.4. Nested Polymerase Chain Reaction

A nested PCR technique was applied in the present study for its sensitivity and specificity. Primer sets designed specifically for *H. pylori* by Song et al. are shown in Table 1. For first PCR reaction, each 50 *μ*L PCR amplification mixture contained 1 *μ*L (1 U/*μ*L) KOD-Plus DNA polymerase High Fidelity (Invitrogen, Japan), 5 *μ*L PCR buffer for KOD-Plus, 1.5 *μ*L of each prime (0.3 *μ*M), 10 ng of genomic DNA, 5 *μ*L dNTPs (0.2 mM each), and 2 *μ*L Mg^2+^ (1.0 mM). For the second reaction, each 50 *μ*L PCR amplification mixture had 1 *μ*L KOD-Plus DNA polymerase High Fidelity (Invitrogen, Japan), 5 *μ*L PCR buffer for KOD-Plus, 1.5 *μ*M of each primer and 1 *μ*L amplicons from the first PCR reaction, 5 *μ*L dNTPs (0.2 mM each), and 2 *μ*L Mg^2+^ (1.0 mM). The thermal cycle conditions were, for the first reaction, 94°C for 2 min followed by 40 cycles of 94°C for 15 sec, 55°C for 30 sec, and 68°C for 1 min and final elongation at 68°C for 4 min and, for the second reaction, 94°C for 2 min followed by 30 cycles of 94°C for 15 sec, 55°C for 30 sec, and 68°C for 1 min and final elongation at 68°C for 4 min. Positive control was conducted with purified *H. pylori* genomic DNA, while ddH_2_O were set as a blank control.

### 2.5. Gel Electrophoresis

The PCR products were visualized with 1.5% agarose gel stained with Goldview (Biohippo, Gaithersburg, USA) under UV gel Imager (Beijing Liu Yi company and vendor location). The PCR product was about 230 bp.

### 2.6. Statistical Analysis

Chi-square test was used for qualitative data, and the Wilcoxon rank test was used for analysing nonparametric (interval or not normally distributed) data. To further exclude the possibility that the statistical significance between the dental and the nondental group was introduced by sampling bias, we stratified enrolled subjects with correlative factors listed above, and the *Z* test was used to compare the detection frequency of *H. pylori* in subgrouped between dentists and nondentists. The logistic regression model was used for correlation analysis. *α* = 0.05 was set as the level of significance, and the statistical analysis was done with SPSS 21.0 and SAS 9.3.

## 3. Results

### 3.1. Overall Included Subject Information

Subjects (*N* = 211, 77 males and 123 females, age: 30 ± 11 years) were recruited in this study. 11 subjects were further excluded due to the absence of either saliva samples or the questionnaire survey. The clinical parameters of enrolled populations are listed in Table 2. No significant difference was observed in the sex and educational background between the dentists and the nondentists (Table 2). However, the percentage of subjects living in an urban area in dentists was higher than that of nondentists (*p* = 0.047, *χ*^2^ = 3.946, Table 2). The dentists enrolled were younger than nondentists (25.52 ± 3.18 vs. 32.76 ± 12.57 years old, *p* < 0.0001, Table 2). In addition, we found that the working years between groups were different (*p* < 0.0001, *Z* = −3.922, Table 2).

### 3.2. Dentists Are at a Higher H. pylori Infection Risk

Approximately 10% (23 out of 200) recruited subjects were detected as salivary *H. pylori* positive, and the prevalence among males was equal to that among females (12.99% vs. 10.57%, *p* > 0.5, *χ*^2^ = 0.272, Table 3). The oral *H. pylori* detection rate in the saliva of dentists was 16.67% (15/90) while 7.27% (8/110) in the control group, and the difference was statistically significant (*p* = 0.038, Table 3). As shown in Table 4, among those with good individual hygiene (e.g., washing hand, never sharing tooth mug, and dishware), healthy lifestyle (e.g., no frequently picky eating, never drink unboiled water and alcohol, and nonsmoking), well physical condition (e.g., no symptom of gastric diseases), no families with gastric diseases, and not having meals with *H. pylori* infection, dentists still showed a higher bacterial detection frequency than the nondentists, which further supported that dentists were more vulnerable to oral *H. pylori* infection (Table 4).

### 3.3. Dental Practice Frequency Is Positively Correlated with the Vulnerability to Oral H. pylori Infection

**Figure 1:**
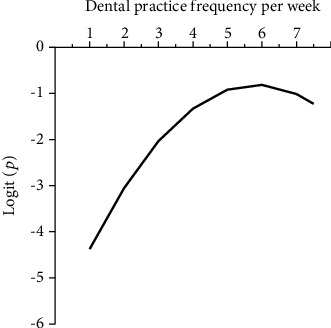
The fitting curve showing the association between the oral *H. pylori* infection possibility and dental practice frequency per week of dentists.

Among using of PPE, dental practice frequency per week (DPF, every half-day defined as one clinical practice), and the number of patients treated every half-day, a correlation between dental practice frequency per week (DPF) and oral *H. pylori* detection rate in saliva was observed (Table 5). The relationship between infection possibility (*P*) and DPF was shown with the equation as the following: logit(*P*) = −5.995 + 1.776 × DPF − 0.152 × DPF^2^. A fitting curve was generated based on the regression equation (Figure 1), and the oral *H. pylori* detection rate increased as the dental service frequency increased.

## 4. Discussion

*H. pylori* infection constitutes a crucial element of the pathogenesis of gastroduodenal ulcer disease, noncardia gastric diseases, and gastroesophageal reflux disease [26–30]. It has also been associated with increased risks of colon cancer [30–32], idiopathic thrombocytopenic purpura [33, 34], iron deficiency anemia [35, 36], vitamin B12 deficiency [37], and more recently, neurodegenerative disorders and metabolic syndrome [38]. Dentists might be particularly prone to *H. pylori* infection, because the oral-oral pathway is one of the primary routes of *H. pylori* transmission. *H. pylori* is able to colonize the oral cavity [18, 21, 39, 40] and is disseminated during dental procedures in the form of “aerosol cloud” [40–42]. Previous studies from the United States, Japanese and French have explored the problem of *H. pylori* infection in dental professionals using serological markers [43–45]. However, studies comparing the detection of salivary *H. pylori* between dentists and nondentists are limited. In this study, we analysed the presence of *H. pylori* in the oral cavity of dentists and nondentists, in the purpose of evaluating the higher risk of *H. pylori* infection in dental professionals.

In this study, saliva was collected, as it is heavily laden with bacteria (10^8^~109 CFU/mL) [46], making it a perfect candidate for *in vivo* oral microbial investigations. Moreover, saliva collection is a noninvasive and easy means for detection compared with other oral sites [47]. The salivary detection rate of *H. pylori* in the current study was much lower than the gastric detection rate in Chinese as reported previously by others (~40%) [48] but was similar to the oral detection rate of *H. pylori* in Mexican asymptomatic children through PCR [49]. We confirmed that the detection frequency of oral *H. pylori* in the dentists was significantly higher than that in the control group (16.67% vs. 7.27%, *p* = 0.038, Table 3). This result was consistent with other investigations [8, 44], supporting that dentists were at a higher risk of *H. pylori* infection. It is noteworthy to mention that some other studies have showed that there were no significant oral *H. pylori* detection differences between dentists and nondentists [50]. This disagreement might be caused by methodologies (culture, serological method, or PCR), geographic factors, etc.

To ensure our research provided an objective and comparable result, subject enrolment was strictly controlled according to the random principle. The demographic characters including the sex and educational background between the two groups were comparable. When it comes to the residence, although majority of both the enrolled dentists and nondentists lived in an urban area, a statistically significant difference was observed. Usually, people living in an urban area have more access to better sanitation in China, which is important to control microbial infection such as *H. pylori* [51–53]. However, the *H. pylori* infection frequency within dentists was significantly higher than that in the control group, supporting the idea that dental practicing increases the *H. pylori* infection risk. We further excluded the influence of confounders as shown in Table 4 by stratifying and compared the *H. pylori* infection between dentist and nondentist subgroups. After controlling these potential biases, dentists still had a higher detection rate of *H. pylori* infection. These results further support the idea that dentists are more vulnerable to oral *H. pylori* colonization and highlight that dental professionals should take these issues seriously.

Furthermore, we analysed factors which might be closely associated with the infection risk among the dentists and tried to provide clues for dentists to take measures to reduce the infection risk. The factors analysed in this study included safety-personal protective equipment (PPE, such as masks, gloves, and gown), dental practice frequency per week (DPF, every half-day defined as one clinical practice), and the number of patients treated every half-day. Among them, DPF was closely related to oral *H. pylori* detection rate, as oral *H. pylori* detection rate grew as the dental practice frequency increased. The equation and the fitting curve were further applied to describe the relationship. The possible explanation was “aerosol cloud” generated during clinical practice can long-time exist [40–42]. Thus, once the dental clinic gets contaminated, without intensive disinfection measures, the increased frequency of exposure will elevate the infection risk. No significant association was observed between PPE and salivary detection rate of *H. pylori*. However, it does not mean that PPE plays no role in preventing oral *H. pylori* transmission, as we noticed that dentists did not wear PPE all the time once they enter the dental clinic. In most cases, they used PPE, especially mask, only when they carried out dental procedures, which may reduce the protective function of PPE. Our data highlights the importance of standard precautions in preventing dentists from oral *H. pylori* infection, including intensive disinfection of the clinic as well as wearing PPE all the time.

## 5. Conclusion

Taken together, the current study confirmed that dental professionals are at a higher risk of *H. pylori* infection compared with nondentists. The frequency of exposure to a dental clinic of the Chinese dentists is the major risk factor for *H. pylori* infection.

## Figures and Tables

**Table 1:** Primers used in the present study.

	Sequence	Product	Refs
Primer 1	5′-CCCTCACGCCATCAGTCCCAAAAA-3′	417 bp	[54, 55]
	5′-AAGAAGTCAAAAACGCCCCAAAAC-3′		
Primer 2	5′-GCCAAATCATAAGTCCGCAGAA-3′	230 bp	
	5′-TGAGACTTTCCTAGAAGCGGTGTT-3′		

**Table 2:** Clinical parameters of study populations.

	Dentists	Nondentist group	Statistical analysis
Age	25.52 ± 3.18	32.76 ± 12.57	*p* < 0.0001^a^
Sex			*p* = 0.174^b^
Male	30 (15.0%)	47 (23.5%)	(*χ*^2^ = 1.845)
Female	60 (20.0%)	63 (31.5%)	
Residence			*p* = 0.047^b^
Urban area	83 (41.5%)	91 (45.5%)	(*χ*^2^ = 3.946)
Rural area	7 (3.5%)	19 (9.5%)	
Educational background			*p* = 0.677^c^
Primary schools and below	0 (0.0)	15 (7.5%)	(*Z* = −0.417)
Junior middle school	0 (0.0)	13 (6.5%)	
Senior school	0 (0.0)	7 (3.5%)	
Junior college education	1 (0.5%)	15 (7.5%)	
Regular college	23 (11.5%)	32 (32.5%)	
Master	55 (27.5%)	22 (11%)	
PhD and above	11 (5.5%)	5 (2.5%)	
Unclear	0 (0.0)	1 (0.5%)	
Years of working			*p* < 0.0001^c^
1-2 yrs	49 (24.5%)	49 (24.5%)	(*Z* = −3.922)
3-5 yrs	34 (17.0%)	31 (15.5%)	
6-10 yrs	6 (3.0%)	11 (5.5%)	
>11 yrs	1 (0.5%)	26 (13%)	
Unclear	0 (0.0)	3 (1.5%)	

^a^ *T*‐*t* test; ^b^chi-square test; ^c^Wilcoxon rank test.

**Table 3:** Detection frequency of oral *H. pylori* in enrolled subjects.

	Negative	Positive	Total	*p* value
Nondentist	102 (51.0%)	8 (4.0%)	110	*p* = 0.038^a^ (*χ*^2^ = 4.292)
Dentist	75 (37.5%)	15 (7.5%)	90	
Male	67 (33.5%)	10 (5.0%)	77	*p* > 0.5^a^ (*χ*^2^ = 0.272)
Female	110 (55.0%)	13 (6.5%)	123	
Total	177 (88.5%)	23 (11.5%)	200	

^a^Chi-square test.

**Table 4:** Detection frequency of oral *H. pylori* in subgrouped volunteers.

	Dentist		Nondentist		*Z* value	*p* value
	Total	Positive	Total	Positive		
Washing hand before eating and after toilet						
Sometimes	2	1 (50.0%)	5	0 (0.0)	1.708	0.088
Always	28	6 (21.4%)	40	2 (0.5%)	2.069	0.039
Every time	60	8 (13.3%)	65	6 (9.2%)	0.727	0.467
Tooth mug sharing						
Never	72	15 (20.8%)	85	5 (5.9%)	2.800	0.005
Sometimes	15	0 (0.0)	15	2 (13.3%)	-1.464	1.000
Always	2	0 (0.0)	2	1 (50.0%)	-1.155	1.000
Every time	1	0 (0.0)	8	0 (0.0)	—	—
Dishware sharing						
Never	38	7 (18.4%)	22	0 (0.0)	2.142	0.032
Sometimes	34	4 (11.8%)	16	2 (12.5%)	-0.075	1.000
Always	12	4 (33.3%)	23	3 (13.0%)	1.424	0.154
Every time	6	0 (0.0)	48	2 (4.2%)	-0.510	1.000
Picky eating						
Sometimes	79	13 (16.4%)	102	7 (6.7%)	2.042	0.041
Always	11	2 (18.2%)	8	1 (12.5%)	0.335	0.737
Drinking unboiled water						
Yes	13	1 (7.7%)	24	2 (8.3%)	-0.068	1.000
No	77	14 (18.2%)	86	6 (7.0%)	2.177	0.029
Smoking						
Yes	2	0 (0.0)	8	2 (25.0%)	-0.791	1.000
No	88	15 (17.0%)	102	6 (5.9%)	2.447	0.014
Alcohol abuse						
Yes	2	0 (0.0%)	4	1 (25.0%)	-0.775	1.000
No	86	14 (16.3%)	106	7 (6.6%)	2.136	0.033
Nausea						
Yes	8	0 (0.0)	12	1 (8.3%)	-0.838	1.598
No	73	14 (19.2%)	87	7 (8.0%)	2.077	0.038
Not sure	6	1 (16.7%)	10	0 (0.0)	1.333	0.182
Gastric diseases						
Yes	21	5 (23.8%)	21	2 (9.5%)	1.242	0.214
No	43	8 (18.6%)	66	3 (4.5%)	2.382	0.017
Not sure	23	2 (8.7%)	21	3 (14.3%)	-0.584	1.000
Families with gastric diseases						
F^a^ (+) M^b^(-)	13	1 (7.7%)	22	3 (13.6%)	-0.534	1.000
F^a^ (-) M^b^ (+)	21	2 (9.5%)	12	2 (16.7%)	-0.605	1.000
F^a^ (+) M^b^ (+)	2	0 (0.0)	3	0 (0.0)	—	—
F^a^ (-) M^b^ (-)	47	12 (25.5%)	69	3 (4.3%)	3.338	0.001
B^c^ or S^d^ (+)	4	0 (0.0)	3	0 (0.0)	—	—
Children (+)	0	0 (0.0)	0	0 (0.0)	—	—
Have meal with *H. p*-positive person						
Yes	2	0 (0.0)	5	0 (0.0)	—	—
No	8	4 (50.0%)	10	0 (0.0)	2.535	0.011
Not sure	45	9 (20.0%)	37	5 (13.5%)	0.777	0.437

^a^Father; ^b^mother; ^c^brothers; ^d^sisters. *Z* test was used to compare the difference between groups. In dishware sharing, nausea, gastric diseases, families with gastric diseases, and families who had meal with *H. pylori*-positive person category, the related information of 1, 2, 4, 5, and 93 subjects was missed, respectively.

**Table 5:** Logistic regression analysis between dental practice frequency per week and oral *H. pylori* infection.

Variable	Partial regression coefficients	Standard error	Wals *χ*^2^	*p* value	OR	OR 95% CI	
						Lower	Upper
Intercept	-5.995	2.382	6.333	0.012	0.002		
DPF	1.776	0.861	4.257	0.039	5.909	1.093	31.946
DPF^2^	-0.152	0.073	4.330	0.037	0.859	0.745	0.991

DPF: dental practice frequency per week.

## Data Availability

The data used to support the findings of this study are available from the corresponding author upon request.
